# Island-mainland song divergence is decoupled from genetic and geographic distance in silvereyes

**DOI:** 10.1093/beheco/arag032

**Published:** 2026-04-09

**Authors:** Marie Robert, Annika Radu, Christine L Dudgeon, Dominique A Potvin

**Affiliations:** School of Science, Technology and Engineering, University of the Sunshine Coast, Sippy Downs, Queensland 4556, Australia; School of Science, Technology and Engineering, University of the Sunshine Coast, Sippy Downs, Queensland 4556, Australia; School of Science, Technology and Engineering, University of the Sunshine Coast, Sippy Downs, Queensland 4556, Australia; School of Science, Technology and Engineering, University of the Sunshine Coast, Sippy Downs, Queensland 4556, Australia; Centre for Bioinnovation, University of the Sunshine Coast, Sippy Downs, Queensland 4556, Australia

**Keywords:** behavior, birdsong, cultural evolution, dialects, island–mainland divergence, bioacoustics, passerine, adaptation

## Abstract

Population connectivity shapes both genetic and cultural variation, including in learned vocal behaviors such as birdsong. Silvereyes (Zosterops lateralis) are well-known island colonizers, providing a useful model for examining how cultural evolution unfolds across populations with differing geographic isolation. We compared song dialects from 4 populations in South East Queensland: 2 mainland (Z. l. cornwalli) and 2 island populations (Z. l. chlorocephalus), for which patterns of genetic connectivity had previously been described. From field recordings, we constructed syllable repertoires and measured spectral features to quantify dialect structure and acoustic divergence. We found no correlation between acoustic distance and either genetic or geographic distance among populations, indicating that dialect divergence is not strongly constrained by neutral genetic structure or spatial separation at this scale. Island populations showed greater similarity to one another, as well as slightly higher syllable diversity, than mainland populations. These patterns are consistent with cultural divergence shaped by local, social, or ecological conditions rather than by geographic or genetic distance. Our findings refine current understanding of island dialect evolution in silvereyes by showing that cultural differentiation can occur despite limited genetic divergence and relatively small geographic distances. Because song is socially transmitted, cultural traits may respond rapidly to environmental change, suggesting that conservation strategies for island passerines may benefit from integrating the cultural dimensions of avian behavior.

## Teaser

Silvereye songbirds in Queensland show surprising differences in their songs across nearby islands and mainland areas. These differences don’t match up with genetic or geographic patterns, suggesting other factors are at play. Island birds had more varied and higher-pitched songs, possibly adapting to their environments. This study shows how bird songs can evolve in unexpected ways and highlights the importance of protecting habitats to preserve cultural traits in wildlife.

## Introduction

Birdsong has long been a topic of interest in animal behavior, yet its inclusion in animal culture research has been inconsistent (Whiten 2021). Because songs are acquired through social learning, transmitted both horizontally (within generations) and vertically (between generations), they provide a powerful system for studying how behavior evolves under the joint influence of environment, social structure and morphology (Kroodsma 1982). In many passerines, songs play key roles in both territory defence and mate attraction, so divergence in song structure can influence mate choice, mate recognition and, ultimately, gene flow and speciation (Mayr 1963; Slabbekoorn and Smith 2002).

Dialects are population-specific variants of song that can develop when socially learned songs diverge among sites. In several species, individuals discriminate between local and foreign dialects and show preferences for local song types, which can bias mating patterns and act as partial barriers to gene flow (eg Colombelli-Négrel and Kleindorfer 2021). This dialectal recognition highlights the interplay between culture and gene flow in speciation (Laland and Janik 2006; Aplin 2019).

Island colonization events provide a powerful context in which to investigate cultural and evolutionary processes, because founder effects, restricted gene flow, ecological shifts and cultural drift can interact over different timescales to shape dialects (Potvin and Clegg 2015; Sendell-Price et al. 2021). The acoustic adaptation hypothesis predicts that songs evolve to maximize transmission in local environments, for example favoring lower frequencies or narrower bandwidths in dense vegetation (Slabbekoorn and Smith 2002). The broader island syndrome framework predicts systematic changes in body size, population density, predation pressure and competition on islands, which can in turn alter selection on vocal signals (Clegg and Owens 2002; Lomolino 2005; Demery et al. 2021). At the same time, colonization bottlenecks and reduced genetic diversity may limit standing variation in both morphology and song (Frankham 1997; Petrie et al. 1998), while relaxed selection on mate choice under reduced genetic diversity could either constrain or relax song elaboration (Griffith 2000; Cardoso et al. 2012).

### Disentangling sources of dialect divergence

Multiple nonmutually exclusive mechanisms may therefore generate geographic song variation. To organize these mechanisms, we follow a 3-timescale framework distinguishing historical–genetic, ecological and cultural processes.

First, historical–genetic processes act on long timescales. Founder effects and reduced genetic diversity on islands may reduce phenotypic variation, which can constrain the outcome of sexual selection and vocal variation (Frankham 1997; Petrie et al. 1998; Griffith 2000), while phylogenetic constraints may preserve ancestral acoustic structure (Porzio et al. 2024). Under this model, we expect greater song similarity among genetically connected populations and a positive relationship between acoustic and genetic distance.

Second, ecological processes act at intermediate evolutionary timescales. The acoustic adaptation hypothesis predicts that song characteristics evolve to maximize transmission in local environments (Hansen 1979), while island syndrome may alter selection on body size, social structure and competition (Clegg and Owens 2002; Lomolino 2005). High population densities and reduced predation on islands may relax constraints on song structure or promote more elaborate signals (Morinay et al. 2013; Doutrelant et al. 2016; Robert et al. 2022). Under this view, island dialects should diverge from mainland counterparts because selection regimes differ.

Third, cultural processes operate on short generational timescales and may lead to rapid, cumulative change in learned songs (Aplin 2019). Cultural drift, biased learning and limited meme flow between populations may generate dialects independently of genetic or environmental divergence. A null cultural model therefore predicts that acoustic distance will not be explained by genetic or geographic distance, and that dialect structure may be population-specific even among connected populations.

Island–mainland comparisons are uniquely suited to separating these mechanisms because cultural exchange is often restricted by water barriers even when genetic divergence remains low (Lachlan et al. 2013; Potvin and Clegg 2015).

### The silvereye system

The silvereye (Zosterops lateralis) is a socially learning passerine native to Australia, New Zealand and surrounding Pacific islands. Silvereyes are notorious island colonizers: they can disperse long distances but become largely sedentary after colonizing islands, and rapidly diversify into distinct subspecies in new habitats (Ottenburghs 2019; Estandía et al. 2023). The selective pressures of geographically isolated environments can accelerate divergent evolution and have likely contributed to the rapid speciation of silvereyes (Sendell-Price et al. 2020). Silvereye song is socially learned but influenced by genetic predispositions (Nelson 2000; Lachlan et al. 2013), and previous work shows that populations adjust their vocal structure in response to urban noise and island colonization (Potvin et al. 2011; Potvin and Parris 2012; Potvin and Clegg 2015).

A recent genetic study of 4 South-East Queensland populations—2 mainland sites, Fraser Coast (FC) and Sunshine Coast (SC), and 2 island sites, Heron Island (HI) and Lady Elliot Island (LEI)—showed that genetic divergence was best explained by water barriers rather than linear distance (Radu et al. 2024). Using genome-wide SNP markers, that study reported pairwise F_ST_ values ranging from 0.02 between the 2 mainland sites to 0.07 to 0.08 between the island populations, and up to 0.19 to 0.21 between island-mainland pairings, indicating substantial genetic connectivity within the mainland and much stronger separation between mainland and island sites. These patterns suggest limited gene flow across water gaps, even at relatively small spatial scales. Whether dialect divergence mirrors this genetic pattern, however, has not been tested.

Silvereyes thus provide an exceptional system for testing the above hypotheses because their repeated, well-documented colonization of offshore islands has produced natural replicates of divergence under different demographic and ecological histories. Vocal learning, rapid cultural change and small founding populations mean that song variation can reflect processes acting over short ecological timescales, while the extensive genetic work on these same populations (eg, Radu et al. 2024) allows historical and demographic influences to be benchmarked. Despite extensive research on silvereye morphology, behavior and genetics, the cultural and ecological processes governing dialect divergence are still not fully understood. Testing these hypotheses in this system therefore fills a critical gap by linking known evolutionary histories with contemporary patterns of vocal culture.

### Study objectives and predictions

Here, we characterize syllable diversity, multivariate spectral structure and dialect similarity among the 4 populations, and test whether acoustic distances correspond to genetic or geographic distances. Building on our 3-timescale framework, we derived the following predictions:

#### Historical-genetic prediction

If song variation is constrained by shared ancestry or connectivity, then acoustic distance should correlate positively with genetic distance, and mainland sites (FC and SC) should be most similar.

#### Ecological prediction

If island ecology shapes song transmission or production, island dialects should diverge from mainland dialects in spectral features or syllable composition, potentially reflecting shifts in habitat, density or competition.

#### Cultural divergence (null) prediction

If dialects evolve primarily through cultural processes such as drift, innovation and limited meme flow, then song divergence should be decoupled from genetic and geographic distances, and each population may express distinct dialect structure despite differing histories.

Song divergence in silvereyes can arise through processes operating on different evolutionary timescales: (i) historical constraints and standing variation inherited from source populations (including founder effects), (ii) post-colonization divergence driven by drift or local ecological adaptation, and (iii) recent cultural transmission processes such as learning, exchange and cultural drift. Because these mechanisms can generate similar patterns of divergence, they can be difficult to disentangle. In this study, our analyses focus on testing patterns that are most consistent with levels (ii) and (iii), while recognizing that level (i)—deep historical constraints—cannot be fully evaluated with the present dataset. We therefore interpret our results with these limitations in mind.

## Material and methods

### Data collection

#### Study sites and species

**Figure 1:**
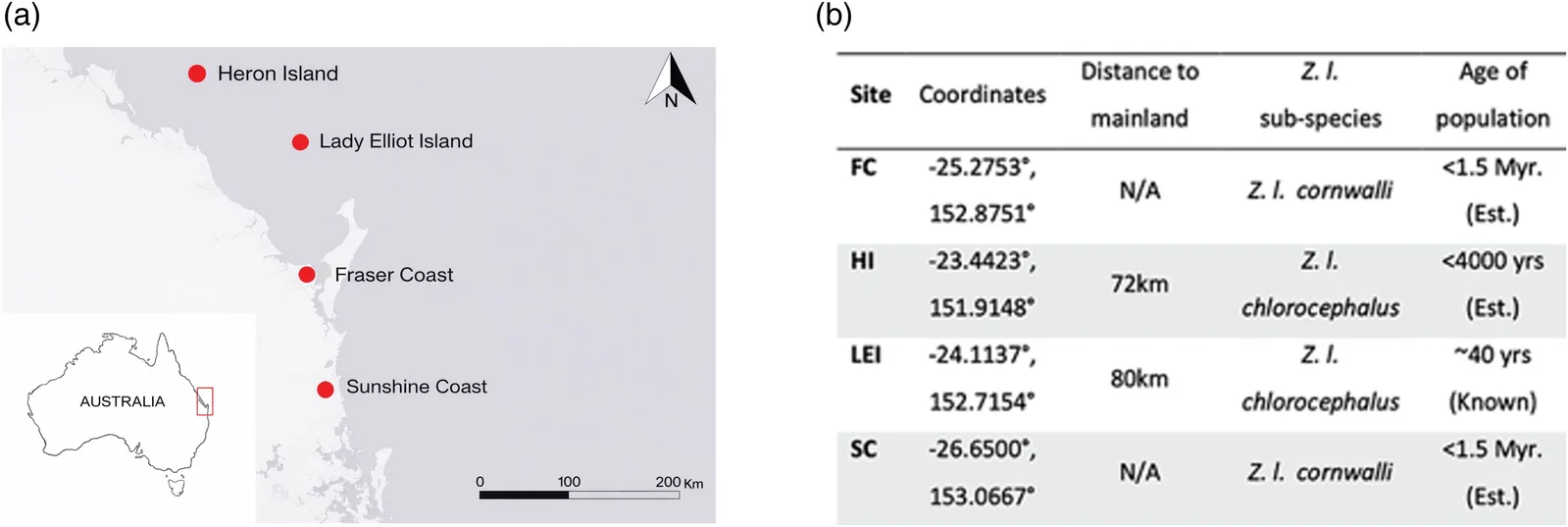
a) Map showing the 4 study sites including 2 mainland sites (SC and FC) and 2 island sites (LEI and HI). Inset highlights the region of study within Australia. b) Biogeographic information for 4 silvereye populations in South East Queensland. Distances from the mainland were measured using the online platform Lexilogos.com. Population age data was extracted from the following literature sources: FC and SC—Estandía et al. (2025); LEI—Degnan (1993); HI—Clegg et al. (2008) and indicated as “known” or “estimated”.

We examined 4 locations in South East Queensland, consisting of 2 island sites, where the subspecies Zosterops lateralis chlorocephalus resides (LEI and HI), and 2 mainland sites, that are inhabited by Z. l. cornwalli (FC and SC). These sites are referred to as LEI, HI, FC, and SC, respectively (Fig. 1). In Radu et al. (2024), the genetic connectivity between the same sites was studied, but the FC site consisted of a single site and was referred to as MB (Maryborough); here, we use FC to reflect multiple recording locations across the FC region, which extends beyond Maryborough.

The findings from Radu et al. (2024) revealed high genetic connectivity between the mainland sites, which could be explained by their geographic proximity and the absence of water barriers. In contrast, the 2 islands showed genetic separation though there was evidence of minimal migration with 1 individual captured at HI having the genetic signature of LEI. The evolutionary ages of these populations have been estimated to be approximately 40 yr (LEI), 4,000 yr (HI) and <1.5 million years (mainland) (Degnan 1993; Clegg et al. 2008; Estandía et al. 2025), thus providing insights into the cultural evolution of dialects at various stages.

We selected these sites based on the background knowledge mentioned above, their geographical proximity, and their approximate equidistance (110 to 154 km), with the island sites similarly distanced from the mainland. This geographic arrangement allows for potential dispersal while representing distinct genetic lineages, making it possible to assess genetic, geographic, and cultural connectivity in a unique manner. Although gene flow between the island and mainland populations is limited, cultural transmission remains a possibility, as visiting silvereyes could introduce new song elements to different populations without contributing to genetic interbreeding.

#### Song data collection

We collected the acoustic data at 48-kHz sampling rate with either a TASCAM DR-40X handheld recorder with a RØDE NTG1 shotgun microphone, or a Marantz Professional PMD660 Solid State recorder with a Sennheiser ME67 directional microphone. We carried out song recording in June 2021 (LEI), February 2021 (SC), and then again in September 2023 to February 2024 (FC, SC, and HI). Although LEI was sampled in June, which precedes the documented breeding season (Sandvig et al. 2017), the population is nonmigratory and showed clear territorial and vocal activity consistent with breeding behavior at the time of recording. These recording timeframes ensured that the resident mainland populations were recorded, as Z. l. cornwalli are known to migrate outside of breeding season while island populations do not (Estandía et al. 2023). Silvereye song production differs between mainland and island populations. Mainland silvereyes produce full song predominantly during the breeding season, with very limited full-song output at other times (Nelson 2000). In contrast, island populations show much weaker seasonality, with year-round production of structurally complete song and more flexible breeding cycles, consistent with patterns documented in island passerines and other Zosterops populations (Potvin and Parris 2012). All LEI recordings consisted of full song indistinguishable from breeding-season vocalisations. Because no nonbreeding vocalization types (eg, subsong or calls) were included in the dataset, and because island silvereyes exhibit extended song production across the year, “season” cannot be treated as a biologically meaningful factor in this analysis. We therefore consider the June LEI recordings functionally equivalent to breeding-season song and do not expect seasonal differences to bias comparisons among sites.

We conducted fieldwork over the course of 5 d per site. We collected data between 04:00 AM and 12:00 PM, at a distance of between 2 and 10 m from the focal individual being recorded. We initiated recordings when a silvereye was located and ended them either when the individual ceased singing, flew away or after a period of 5 min of constant song. This approach is consistent with previous studies on avian song recordings to ensure comparability across samples through consistent data collection (eg, Potvin and Clegg 2015). For each recording, we documented the location, date, and time of day. Silvereyes have small territories of up to a 32 m radius during the breeding season (Catterall et al. 1989), hence we recorded individuals from different perching locations separated by at least 70 m to reduce the chance of recording the same individual multiple times. We recorded 6 to 18 individuals per study site (FC: 6, HI: 18, LEI: 11, SC: 11), for a total of 46 individuals. The lower sample size at FC (6 individuals) compared with the other sites (11 to 18 individuals) may have contributed to increased sensitivity to outliers, and this was considered when interpreting patterns of acoustic divergence.

### Data processing

#### Syllable extraction and classification

We generated spectrograms of recordings and measured song variables using RavenPro 1.6 software (Cornell Lab of Ornithology 2011, Ithaca, NY, USA: an example of a silvereye song and syllable spectrogram is provided in Supplementary Material S1). We visually selected individual syllables using the program's in-built selection tool and subsequently categorized them based on their structural similarity. We classified a total of 254 syllable types with distinct spectral structures using this process. Manual selection and categorization of syllables offers numerous advantages over automated processes (Jones et al. 2001). However, it can introduce subjectivity in the consistency and accuracy of classification. To account for this, all syllables were manually classified by a single observer (MR) following fixed criteria to ensure internal consistency.

For each analysis, we selected the classification level appropriate to the scale of structure being evaluated: L0 for fine-scale variants, L1 for intermediate types and L2 for coarse architectural categories (Supplementary Material S2). Because L2 collapses distinct L0 and L1 structures into only a few broad shapes, it risks obscuring biologically meaningful variation; accordingly, only L0 and L1 were used for all inferential analyses (diversity metrics, distance measures and mixed models). L2 was used descriptively to illustrate coarse repertoire structure only.

Automated clustering methods can be valuable for large exploratory datasets, but they often group syllables by acoustic similarity in ways that do not map cleanly onto biologically meaningful categories such as dialect-level types. These were further precluded by the amount of heterogeneity in background noise and recording conditions in our dataset. Given our focus on dialect structure and our moderate sample size, we prioritized a transparent, observer-defined hierarchy (L0 to L2), supplemented by multivariate analyses (Gower distance, PCA, PERMANOVA and mixed models, see below) to quantify acoustic and compositional divergence. This provided a robust operational framework that captured meaningful structural differences while avoiding artifacts introduced by automated clustering. By evaluating population-level dialect differences using multilevel categorical structures, we were able to detect divergence arising from post-colonization processes or recent cultural transmission, even if deeper historical relationships among syllable lineages may not be resolved.

We created 6 datasets for use in further analyses in the R statistical environment (R Core Team 2024) (Supplementary Material S3 and S4), through the creation of both individual and population-level repertoires (ie the number and types of all syllables sung). Figures based on these datasets were created using ggplot2 (v3.5.0) (Wickham 2016). For each level of classification (L0, L1, L2), syllable use was recorded in 3 formats: raw occurrence counts (eg, L0Occ), proportional use expressed as a percentage of total syllables (eg, L0Perc), and binary presence/absence (eg, L0Bin). Additional datasets were created to adjust for sample size differences between sites (L1Adj), and a separate acoustic dataset incorporated the extracted spectral features (Supplementary Material S5) alongside metadata such as site, habitat type, track name, and classification level.

#### Spectral features and multivariate structure

We directly extracted 8 spectral characteristics from each syllable unit in RavenPro: high frequency (Hz), low frequency (Hz), peak frequency (Hz), center frequency (Hz), delta time (s), average slope (Hz/ms), frequency 25% (Hz), frequency 75% (Hz). We obtained bandwidth (Hz) by subtracting low frequency from high frequency. We calculated mean frequency (Hz) by adding the values of low and high frequencies and then dividing the sum by 2 (See Supplementary Material S5 for descriptions). These spectral features are accepted as relevant units for the quantitative and comparative analysis of bird songs (Stowell and Plumbley 2014; Oswald et al. 2022; Amador and Mindlin 2023).

**Figure 2:**
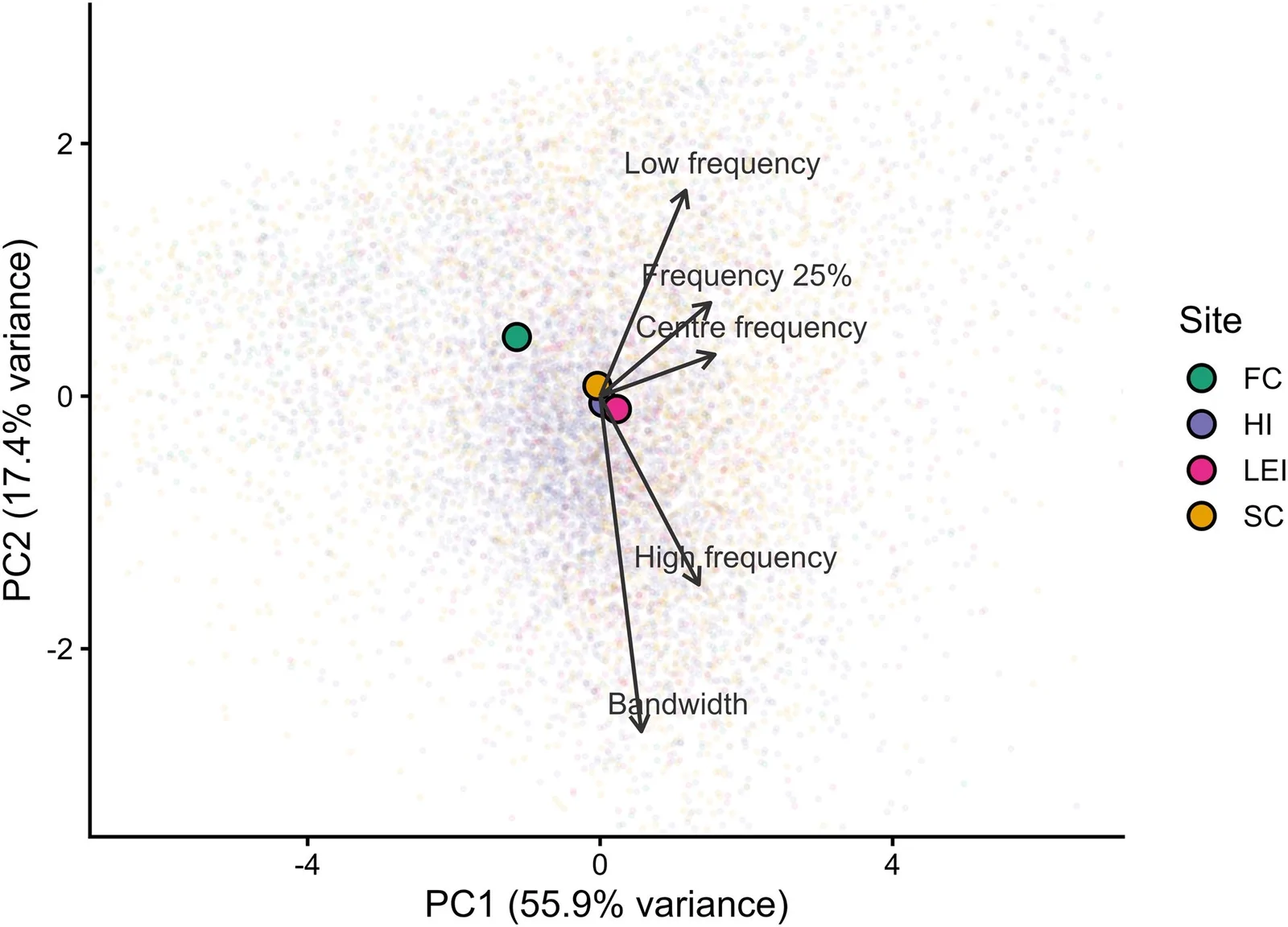
PCA of multivariate spectral features of silvereye syllables across the 4 study sites (FC, SC, HI, LEI). Scores for all syllables (*n* > 10,000) are shown on the first 2 principal components (PC1 = 55.9% variance; PC2 = 17.4% variance). Points represent individual syllables, colored by site, with semi-transparent plotting to illustrate density. Larger circles indicate centroids for each site, with arrows showing the direction and effect of spectral features on each principal component.

To describe the multivariate structure of syllable acoustics and avoid treating these interdependent variables as independent, we conducted a global principal component analysis (PCA) on the eco-acoustic dataset (all sites combined), using centered and scaled values of the 10 spectral variables (HF, LF, PF, CF, DT, Sl, F25, F75, MF, Bw). PCA was performed with the prcomp function in R. We retained the first 3 principal components (PC1 to PC3), which together explained 84.1% of the total variance in spectral features (see Results). For each site (FC, HI, LEI, SC), we calculated the centroid in PC space (mean PC score across all syllables belonging to that site). We then computed Euclidean distances among site centroids in PC1 to PC3 space, obtaining a matrix of multivariate spectral distances between populations. We used this spectral distance matrix in 2 ways. First, it allowed us to visualize patterns of multivariate divergence among sites (Fig. 2), and second, it gave an acoustic distance matrix to be used for Mantel tests relating vocal divergence to genetic and geographic distances (see “Dialect comparisons and distance matrices” below).

We also used ANOVAs on individual spectral variables (eg, mean frequency, delta time) as complementary tests of specific, interpretable hypotheses (such as island–mainland contrasts), but we interpreted these in the context of the multivariate PCA and did not treat them as fully independent tests.

#### Syllable diversity estimation and quantification

We utilized dataset L0Occ (see Supplementary Material S3) to obtain measures of syllable diversity. We chose Simpson's reciprocal index (1−D) as a measure of repertoire diversity because it accounts for both the richness and evenness of syllable types in the dataset, giving more weight to common or dominant syllables (Magurran 2021; Zhao et al. 2022). This makes it particularly suitable for datasets where syllables may be disproportionately represented. Although Simpson's index is more commonly used as a measure of biodiversity, it has also been applied in the context of birdsong repertoire diversity (eg, Shishkina 2020) to effectively capture these dynamics. We chose Shannon entropy (H) to gain insights into the evenness of the distribution of syllables in a site repertoire (Shannon 1948; Sawant et al. 2022), offering a complementary perspective to the richness-focused measure provided by Simpson's reciprocal index. We then normalized the Shannon entropy values to enhance the clarity and interpretability of the results (H′). The following formulas were used for these calculations:

**Table:** 

Simpson reciprocal index (1−D)	Shannon entropy value (H)	Normalized H values (H′)
$1-D=1-\sum {\left(\frac{\;p_{i}}{N}\right)}^{2}$	$H=-\overset{N}{\underset{i=1}{\sum }}\;p_{i}\times \ln\;(\;p_{i})$	$H^{\prime }=\frac{H}{\log_{2}(n)}$

Where:

*p_i_* = proportion of syllables belonging to the *i*th syllable (relative abundance of each syllable).

*N* = total number of syllable occurrences in the population.

*n* = number of distinct syllable structures in the dataset (for L0: *n* = 254; for L1: *n* = 46, for L2: *n* = 14).

To estimate and compare syllable diversity in the 4 populations while accounting for sample size discrepancies, we selected statistical approaches of rarefaction and extrapolation which are commonly used for such estimates (Gotelli and Colwell 2001; Chao et al. 2014). We computed these curves using the iNEXT online software (Chao et al. 2016). We conducted a sample-size based calculation on the datasets L1Occ and L1Adj (Supplementary Material S3). Both curves were generated using the Shannon diversity index with 50 bootstrap replications and 95% confidence intervals.

#### Dialect comparisons and distance matrices

##### Gower distances in syllable composition

The Gower index has proven to be an efficient approach to evaluate the distinctions among dialects across various populations (Potvin and Parris 2012; Potvin and Clegg 2015; Potvin et al. 2019). We chose this index for its pertinence for abundance-based data, its capability of accommodating various sample sizes, and its ability to handle large amounts of zero values (Potvin et al. 2019), characteristic of syllable datasets.

We generated dissimilarity matrices to quantify the extent of syllable sharing between the study sites at different levels of classification using datasets L1Perc, L1Bin, and L1Adj (Supplementary Material S3). We calculated Gower distances in R using the daisy function in the cluster package (v2.1.6) (Maechler et al. 2023). These matrices were used to describe syllable composition differences among the 4 sites and test for island–mainland differences in syllable composition using permutational multivariate analysis of variance (PERMANOVA; see below).

##### Analysis of syllable ranking consistency

To directly test whether populations agree on which syllable types are most versus least common, we analysed the ranking of syllable proportions across sites. For each site, we calculated the proportion of total syllables represented by each L1 syllable type. We then ranked syllables within each site from most common (rank 1) to least common.

We assessed ranking consistency using 3 complementary approaches. First, we used Kendall's coefficient of concordance (W) to test whether the ranking of syllables (most commonly used to least commonly used) is consistent across all 4 sites. W ranges from 0 (no agreement) to 1 (perfect agreement), with intermediate values indicating partial consensus. Second, we calculated pairwise Spearman rank correlations between the syllable rankings of each pair of sites to assess the strength and pattern of pairwise similarity. Finally, for each possible pair of syllable types, we determined whether their relative ordering (which is more common) was consistent across all sites. The percentage of syllable pairs maintaining consistent ordering provides an intuitive measure of overall consensus.

We also conducted a region-level comparison by calculating the correlation between mean syllable proportions in island (HI + LEI) versus mainland (SC + FC) populations.

These analyses directly address the question of whether populations show cultural consensus in syllable usage preferences, independent of differences in overall abundance or repertoire size.

#### Acoustic, genetic and geographic distances and Mantel tests

To explore the association between acoustic distance, geographic distance, and genetic distance, we computed 3 sets of pairwise dissimilarities among the 4 sites. Spectral acoustic distance was obtained from the PCA described above, as the Euclidean distance between site centroids in PC1 to PC3 space (multivariate spectral distance). Genetic distance was provided by pairwise F_ST values from Radu et al. 2024. Geographic distance (km) was measured using the Haversine index by providing precise latitude and longitude data from each site in the R package geosphere (v1.5-18) (Hijmans 2022). We used the acoustic distance matrix and each of the other matrices in Mantel tests conducted with the R package vegan (2.6 to 4) (Oksanen et al. 2022), using the Pearson correlation coefficient and up to 9,999 permutations (limited by the number of unique permutations available for 4 sites). These tests assessed the strength and statistical significance of associations between multivariate spectral distance and (i) genetic distance, and (ii) geographic distance.

In addition, we conducted Mantel tests using Gower distance matrices derived from syllable composition at L0 and L1 (datasets L0Perc, L1Perc, and L1Adj; Supplementary Material S3) to evaluate whether patterns of syllable sharing showed similar or contrasting relationships with genetic and geographic distance.

#### PERMANOVA: island–mainland effects

To test whether acoustic structure differed systematically between island and mainland populations, we conducted PERMANOVAs using the adonis2 function in vegan. For multivariate spectral structure, we used the Euclidean distance matrix derived from PC1 to PC3 scores of individual syllables (eco-acoustic dataset) and modeled the effect of region (Island vs Mainland). For syllable composition, we used the L1Adj Gower distance matrix and modeled region in the same way. In both cases, significance was evaluated with permutation tests (up to 9,999 permutations, again constrained by the number of unique permutations available), and we report *F* statistics and *R*^2^ as effect size measures.

## Results

### Syllable diversity

At level L0 of syllable classification, 254 syllables with unique structures were identified from a total of 10,552 syllables. Of these, 77 were used across all 4 sites. Diversity calculations can be found in Table 1.

**Table 1:** Sampling information of acoustic data for 4 silvereye populations in South East Queensland.

Site	L0 values				L1 values	
	Total syllables recorded	Observed syllable diversity	Shannon Diversity Index (H′)	Simpson's Reciprocal Index (1−D)	Shannon Diversity Index (H′)	Simpson's Reciprocal Index (1−D)
FC	342	101	0.523	0.978	0.564	0.925
HI	5,756	223	0.595	0.987	0.621	0.958
LEI	1,127	170	0.572	0.985	0.603	0.953
SC	3,327	208	0.574	0.984	0.585	0.941

Syllable diversity is sample-size based; Chao2 95% confidence interval (CI) is an estimate accounting for unsampled syllables. Measures were calculated using dataset L0Occ and L1Occ (respectively for L0 and L1). Shannon diversity index indicates normalized results (H′). Shannon diversity and Simpson's reciprocal index values closer to 1 indicate higher diversity.

**Figure 3:**
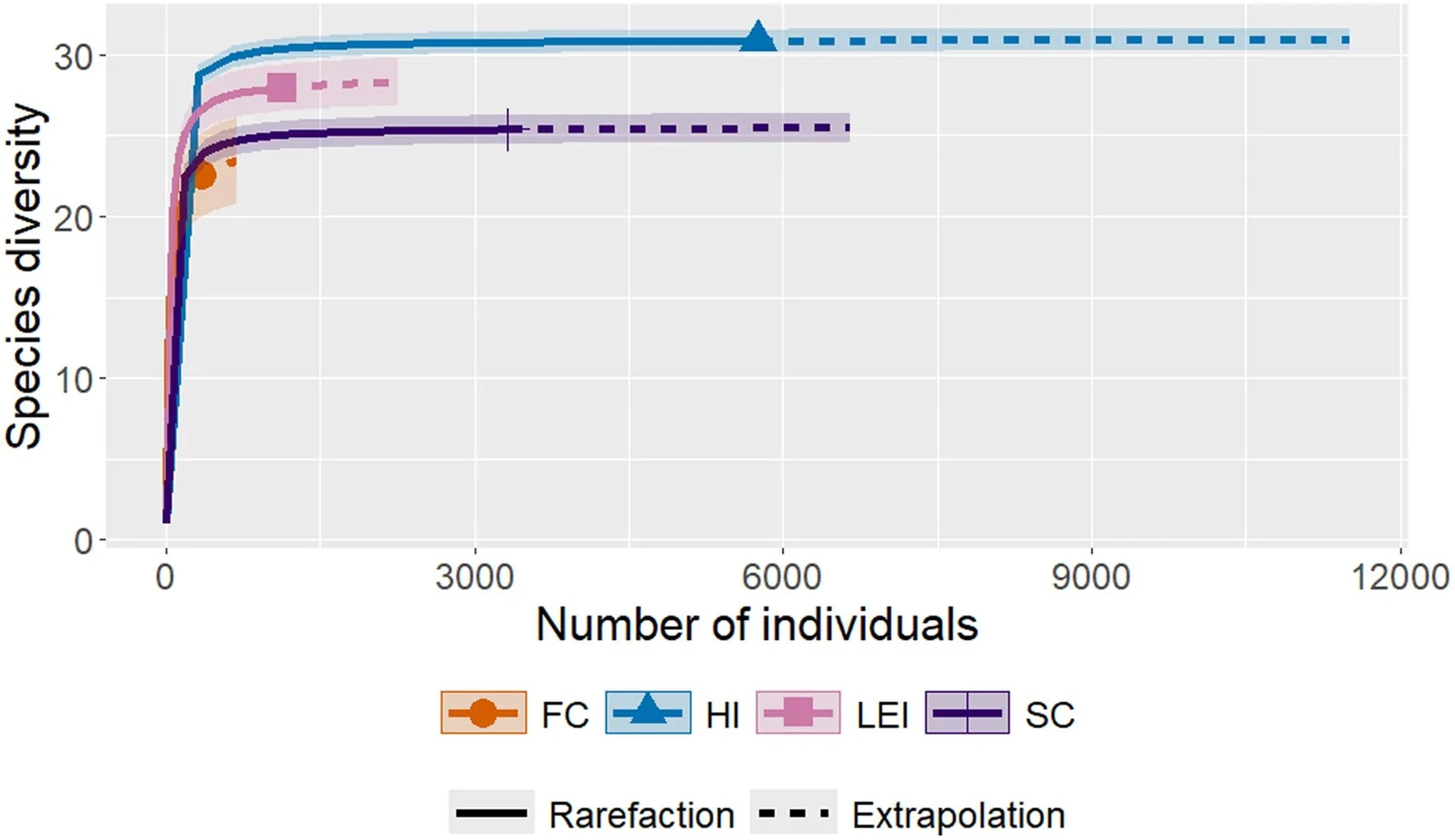
Sample-size based rarefaction-extrapolation curve of syllable diversity in 4 silvereye populations, computed using Shannon diversity index. Solid lines represent observed samples. Polygons represent sample size reference point and change from rarefaction to extrapolation curve. Extrapolation is represented by dotted lines. Confidence interval bands surrounding the lines represent the standard error of the curves. Curve computed using occurrence-based dataset L1Occ.

**Figure 4:**
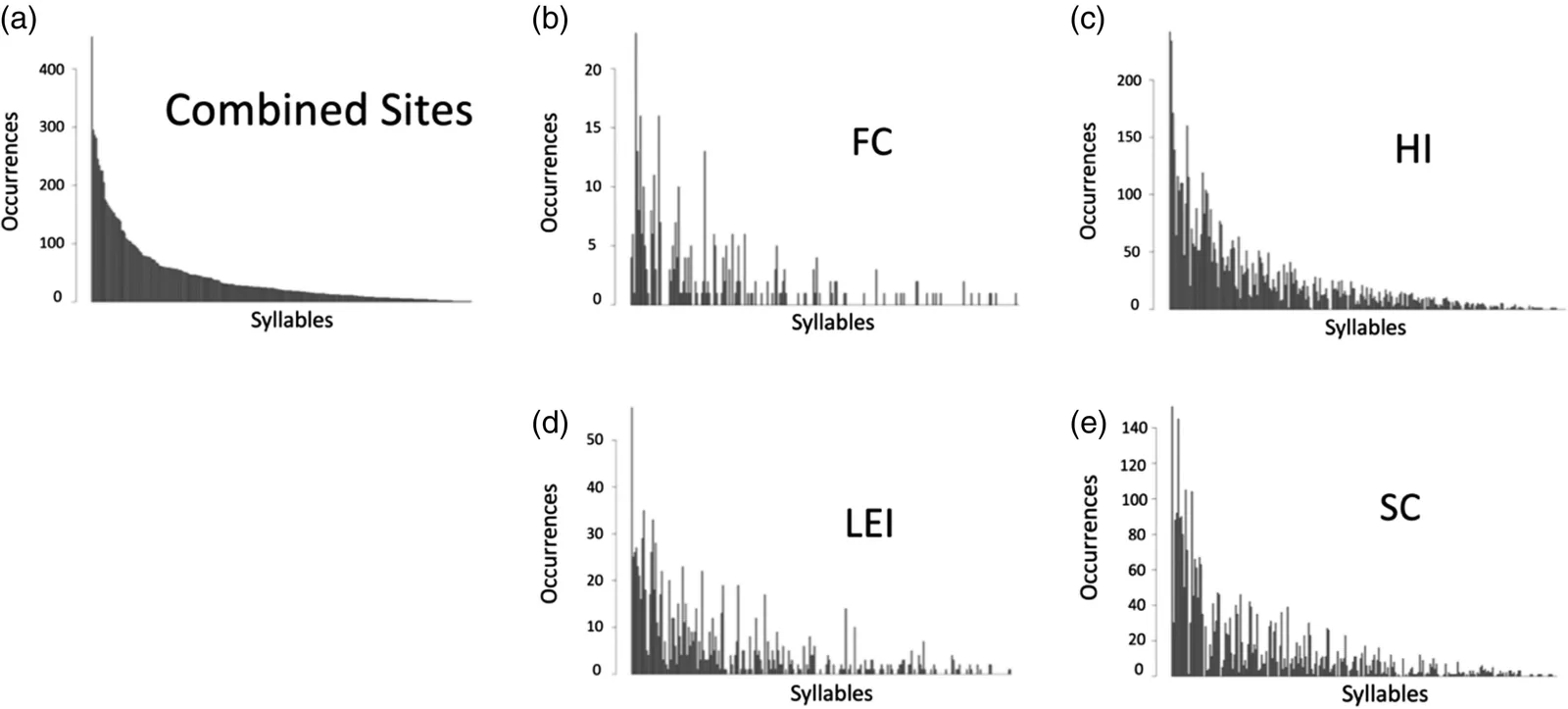
Syllable distribution patterns across the 4 silvereye populations at syllable classification level L0. HI, Heron Island; LEI, Lady Elliot Island; FC, Fraser Coast; SC, Sunshine Coast. Sample sizes per site: FC = 6 individuals, HI = 18, LEI = 11, SC = 11. a) Combined syllable occurrences across all sites, plotted from most to least frequent syllable (global rank order). b–e) Site-specific syllable usage plotted in the exact same syllable order as in panel (a), allowing direct comparison of how each ranked syllable is used across sites. The *x*-axis therefore represents a fixed list of syllable types (global rank), and the *y*-axis indicates the number of occurrences per site. Histograms computed with dataset L0Occ.

At syllable classification level L0, the values obtained for observed diversity, Simpson's reciprocal index (1−D) revealed more diversity on the island sites (HI and LEI). At syllable classification level L1, Simpson's reciprocal index (1−D) and Shannon index (H′) indicated a more diverse and evenly distributed repertoire composition on island sites (HI and LEI) and a lower diversity on mainland sites (FC and SC). A rarefaction-extrapolation curve computed from dataset L1Occ illustrates these findings and predicts continuity in this rank as sampling efforts increase, with maintained higher levels of diversity for island populations (HI and LEI) compared with mainland populations (FC and SC) (Fig. 3). At both syllable classification levels and across all diversity measures, HI consistently exhibited greater diversity than LEI (Fig. 3). Although numerical differences in diversity indices are modest, they are consistent across indices and classification levels (Fig. 4).

Gower index measures of acoustic distance all indicated comparable patterns across datasets, with the highest similarity observed between the 2 island sites, HI and LEI (L1Perc: *G* = 0.46; L1Adj: *G* = 0.44; Supplementary Materials S5 to 8). In contrast, all site pairs involving FC possessed the highest levels of cultural dissimilarity. The mainland-island pair FC–HI displayed the greatest acoustic distance (L1Perc: *G* = 0.68; L1Adj: *G* = 0.68). The L1Bin dataset, which retains only presence–absence information, produced systematically lower overall distance values (eg, HI–LEI: *G* = 0.02; FC–HI: *G* = 0.17) due to the loss of abundance structure, but it nevertheless preserved the same relative ranking of dissimilarity among site pairs. These results were fully consistent across analytical datasets (L1Perc, L1Adj; Supplementary Material S8).

A PERMANOVA on the L1 Gower distance matrix with region as a factor (island vs mainland) did not detect a significant effect of region on syllable composition (*F*_1_,_2_ = 0.54, *R*^2^ = 0.21, *P* = 0.67). Because this analysis uses only 4 site centroids (*n* = 4), statistical power is inherently low; nevertheless, the results indicate that island–mainland differences in syllable composition could not be confirmed statistically at this spatial scale. Overall, the diversity indices and distance patterns suggest that island sites are more similar to each other in syllable composition than to mainland sites, and that FC in particular is acoustically distinct, but that these differences are subtle when based on syllable composition alone.

### Spectral features

Of the 10 spectral features examined in an analysis of variance (ANOVA), 6 exhibited statistically significant associations with habitat type (mainland vs Island) (Table 2). Notably, mean frequency was significantly higher for island populations (mean 3,947 Hz) than for mainland populations (mean 3,878 Hz). Syllable duration was also significantly longer on islands, with a mean delta time value of 0.138 s on islands and 0.109 s for mainland populations.

**Table 2:** Statistical significance values of 10 spectral features of silvereye syllables examined in an ANOVA in relation to habitat type (island vs mainland), and mean values of each characteristic per site (FC, HI, LEI, and SC).

	Statistical values island vs mainland			Mean values per site			
	*F* value	df	*P*-value	Mean FC	Mean HI	Mean LEI	Mean SC
Mean frequency (MF)	68.74	1	**<2e−16**	3,678 Hz	3,961 Hz	3,957 Hz	3,898 Hz
Delta time (DT)	629.6	1	**<2e−16**	0.10 s	0.14 s	0.13 s	0.11 s
Low frequency (LF)	55.94	1	**8.07e−14**	3,038 Hz	3,193 Hz	3,137 Hz	3,119 Hz
High frequency (HF)	42.57	1	**7.14e−11**	4,319 Hz	4,730 Hz	4,777 Hz	4,678 Hz
Center frequency (CF)	8.823	1	**0.00298**	3,698 Hz	3,878 Hz	3,917 Hz	3,875 Hz
Frequency 25% (F25)	16.53	1	**4.83e−05**	3,508 Hz	3,673 Hz	3,706 Hz	3,658 Hz
Average slope (Sl)	2.718	1	0.0993	−0.07 Hz/ms	−1.63 Hz/ms	−1.82 Hz/ms	−2.15 Hz/ms
Peak frequency (PF)	0.766	1	0.381	3,716 Hz	3,873 Hz	3,951 Hz	3,894 Hz
Frequency 75% (F75)	0.467	1	0.494	3,884 Hz	4,087 Hz	4,140 Hz	4,110 Hz
Bandwidth (Bw)	2.123	1	0.145	1,281 Hz	1,537 Hz	1,640 Hz	1,559 Hz

Bold indicates significant p-values (< 0.05).

Other acoustic characteristics that differed significantly between habitat type were low frequency, high frequency, center frequency, and frequency 25%. The 4 variables average slope, peak frequency, frequency 75%, and bandwidth showed statistical significance in their correlation to specific sites (FC, HI, LEI, SC) but not with habitat type (mainland vs island; see Supplementary Materials S9 and S10).

Because several variables were strongly correlated, we summarized spectral structure using PCA rather than multiple univariate tests (see Supplementary Material S11).

A global PCA on the suite of spectral features measured from all syllables across all 4 sites revealed that the acoustic variation was concentrated in a few primary dimensions. Specifically, the first 3 principal components (PCs) together captured 84.1% of the total multivariate variance (PC1: 55.9%, PC2: 17.4%, PC3: 10.8%). PC1, which explained the majority of variance, was primarily driven by features related to average frequency and syllable duration, with higher PC1 scores corresponding to syllables with higher mean frequency and longer duration (Fig. 2). The positions of site centroids in this reduced acoustic space showed clear separation. FC occupied the lowest values on PC1, while the island sites HI and LEI showed the highest PC1 scores. SC was intermediate. Pairwise Euclidean distances between site centroids in PC1 to PC3 space confirmed this pattern, ranging from 0.24 (between the 2 island sites, HI–LEI) to 1.48 (between the mainland FC and island LEI). This indicates that island sites are acoustically more similar to each other than to mainland sites and that FC is particularly distinct.

A PERMANOVA on the Euclidean distance matrix of individual syllables in PC1 to PC3 space detected a statistically significant, albeit small, effect of region (island vs mainland) on multivariate spectral structure (*F*_1_,_10550_ = 46.7, *R*^2^ = 0.004, *P* = 0.001). With more than 10,000 syllables, this indicates that island and mainland syllables occupy slightly but consistently different regions of acoustic space: island syllables tend to be higher in frequency and longer in duration, while mainland syllables (particularly from FC) tend to be lower in frequency and shorter in duration.

### Dialect similarities and relationships with genetic and geographic distance

#### Dialect similarities and syllable usage

**Figure 5:**
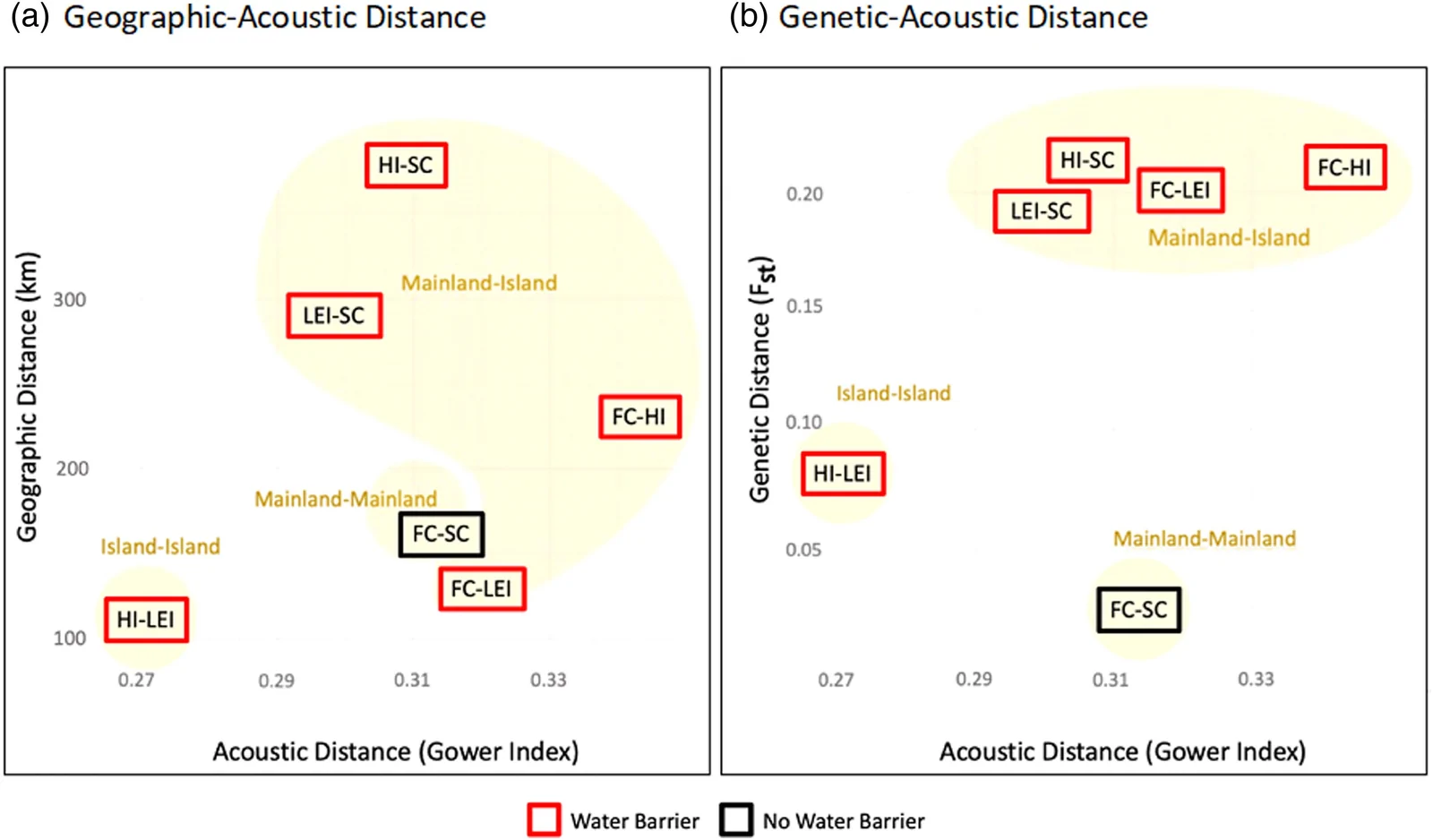
Scatter plots showing relationships between acoustic distance and a) geographic distance, and b) genetic distance, in 4 silvereye populations in South East Queensland (6 site pairs). Acoustic distance was computed from dataset L1Adj using the Gower index. Geographic distance was measured using the Haversine formula, and genetic distance was based on F_ST_ values (Radu et al. 2024). Yellow background shading was manually added to highlight general groupings among site pairs (island–island, mainland–mainland, mainland–island) as distance values increase. It is intended as a visual aid only and does not represent modeled or statistically derived regions. Red squares represent site pairs separated by a water barrier; black squares indicate no water barrier in a site pair. Mantel tests (see Results) showed no significant correlation between acoustic distance and either genetic or geographic distance, indicating that populations that are genetically or geographically close do not necessarily share more similar dialects.

Gower distance patterns (Supplementary material S8) and spectral distances (Table 2, Fig. 5) both indicated that island sites share more similar syllable composition and spectral structure with each other than with mainland sites, and that FC is the most divergent population. Our multivariate analyses (Gower distances, PCA) revealed patterns of syllable sharing and acoustic divergence among populations. To complement these, we examined whether sites showed consistent patterns in which syllable types were most versus least common. Kendall's coefficient of concordance revealed strong overall agreement in syllable rankings across all 4 sites (*W* = 0.747, χ^2^(45) = 134.5, *P* < 0.001), indicating substantial consensus in which syllables tend to be relatively common versus rare. Pairwise Spearman correlations showed moderate average similarity between site rankings (mean *ρ* = 0.666, range: 0.581 to 0.745), with the strongest agreement between HI and LEI (*ρ* = 0.745) and weakest between FC and LEI (*ρ* = 0.581). Despite this overall agreement, only 52.1% of syllable pairs (539 of 1,035 possible pairs) maintained consistent ordering across all sites. This suggests that while populations show strong consensus regarding the most common and rarest syllable types, there is substantial reshuffling of middle-ranking syllables.

Region-level comparison revealed strong correlation between island and mainland syllable proportions (*ρ* = 0.712), indicating that the broader island-mainland distinction does not override shared patterns of syllable preference.

#### Relationships between acoustic, genetic, and geographic distances

Using Gower distance matrices derived from syllable composition, Mantel tests detected no significant correlation between acoustic–genetic or acoustic–geographic distances for any dataset. For the geographic-acoustic comparison, results were nonsignificant for the L0Perc (Mantel statistic *r* = 0.167, *P* = 0.1667), L1Perc (*r* = −0.970, *P* = 1), and L1Adj (*r* = −0.574, *P* = 1) datasets. Similarly, for the genetic-acoustic comparison, results were nonsignificant for L0Perc (*r* = −0.237, *P* = 0.5), L1Perc (*r* = −0.235, *P* = 0.667), and L1Adj (*r* = 0.459, *P* = 0.667). Thus, populations that are genetically similar or geographically proximate do not necessarily possess more similar syllable repertoires (Fig. 5).

Similarly, Mantel tests using multivariate spectral distances from the global PCA detected no significant correlation between spectral and genetic distance (Mantel *r* = 0.052, *P* = 0.42). Although the Mantel test between spectral and geographic distance was also not statistically significant (*r* = −0.41, *P* = 0.71), the moderate negative correlations here and above is noteworthy. This pattern suggests a potential trend where spectral similarity may decrease with geographic proximity among these populations; however, this relationship remains statistically unconfirmed and requires further investigation with greater power.

Taken together, the mixed-model, PERMANOVA, and Mantel results indicate that island and mainland populations differ in dialect structure and multivariate spectral properties, but that these differences are not well explained by neutral genetic divergence or by simple geographic distance.

## Discussion

This study challenges conventional notions of avian dialect formation on islands by showing that dialect divergence in silvereyes is not well predicted by genetic or geographic distance, either in syllable composition or in multivariate spectral structure. Instead, our findings suggest that multiple evolutionary processes interact to shape silvereye vocal behavior, supporting aspects of cultural evolution, acoustic adaptation, island syndrome, and character release.

Across all analyses, 3 main patterns emerge. First, island populations show slightly but consistently higher syllable diversity than mainland populations. Second, syllable usage and multivariate spectral structure differ among sites and between island and mainland regions, although the island–mainland effect on spectral structure is small. And finally, neither genetic relatedness nor straight-line geographic distance predicts acoustic divergence.

Together, these patterns suggest that local cultural processes (learning, drift, innovation) and habitat-specific selection shape dialect divergence, rather than dialects mirroring neutral genetic structure or geographic separation.

### Syllable diversity

Under the historical-genetic prediction (Prediction 1), syllable diversity might reflect shared ancestry, whereas under the ecological or cultural predictions (Predictions 2 to 3), diversity could differ among sites. Our results show that syllable diversity was consistently higher on the island sites (HI and LEI) than on the mainland sites across both syllable dominance (L0) and dialect structure (L1) classification levels. At L0, where the Simpson's reciprocal index reflects syllable dominance and the probability that 2 randomly chosen syllables belong to different categories, this increased diversity may be linked to social dynamics within isolated populations, such as reduced intraspecific competition and less aggressive behavior towards conspecifics (Morinay et al. 2013). Such conditions can foster a more even distribution of syllable usage, resulting in fewer syllables dominating the repertoire and ultimately contributing to higher Simpson diversity. At L1, which emphasizes dialect differences, island sites again demonstrated the highest syllabic diversity, showing greater richness, evenness, and fewer dominant syllables than mainland sites.

While both island sites exhibited high diversity, HI consistently demonstrated greater diversity than LEI at all syllable classification levels and across every diversity measure. Factors associated with insularity, such as silvereyes’ propensity to innovate after colonizing an island (Potvin and Clegg 2015) or the reduced predation on these sites (Gavriilidi et al. 2022), may contribute to the development of more diverse vocal repertoires on islands compared with mainland sites. This pattern is analogous to character release observed in other behaviors or traits on islands, where reduced predation pressure and inter-specific competition allow for greater ecological and behavioral flexibility (Mayr 1963).

Our findings align with broader patterns of acoustic character release on islands, where reduced predation and inter-specific competition can promote greater behavioral and acoustic variation (Mayr 1963; Gavriilidi et al. 2022). In silvereyes, this may be further amplified by occasional dispersal from mainland populations, which could introduce novel syllables and counteract cultural bottlenecks following island colonization (Estandía et al. 2025). Although song exchange between island and mainland passerines is poorly documented, such input could enrich island repertoires without homogenizing local dialects.

Conversely, strong vocal divergence between nearby mainland and island populations (eg, FC and HI) could reflect behavioral reinforcement rather than geographic isolation. Observations of aggression by island silvereyes towards mainland conspecifics (S. Clegg and D. Potvin, personal communication) are consistent with reproductive character displacement (Brown and Lemon 1979), where distinct dialects serve as a premating barrier to prevent interbreeding. Similar acoustic partitioning has been observed in other bird systems where genetic connectivity persists despite cultural divergence (eg Pavlova et al. 2012).

Under our historical-genetic prediction, we expected the 2 mainland sites (FC–SC) to be the most similar, reflecting the genetic connectivity previously established (Radu et al. 2024), followed by island site pair HI-LEI, representing the same subspecies. While acoustic similarities between HI-LEI were confirmed, mainland sites FC-SC showed a moderate to high level of acoustic divergence, indicating that the historical-genetic prediction was not supported. This divergence is surprising given their genetic connectivity and may be attributed to subtle site-specific environmental differences—such as habitat structure or ambient noise—that can influence vocal adaptation, even within the same geographic category (ie, mainland). Social dynamics (eg, differences in territorial behavior) may also affect vocal behavior more strongly than genetic factors alone.

According to our prediction framework, acoustic divergence should correlate with geographic distance only if geographic isolation limits cultural exchange. We tested this by evaluating correlations between acoustic and geographic distance using Mantel tests. No significant correlations were found at either the syllable-composition or spectral-structure levels, consistent with the cultural divergence prediction, in which dialect evolution is decoupled from geographic separation. The similarity between HI–LEI may therefore reflect either acoustic adaptation to similar habitats or relatively recent shared ancestry. Moreover, although HI and LEI are geographically the closest pair (∼110 km), the absence of a correlation between distance and acoustic similarity indicates that this proximity does not provide a general explanation for acoustic resemblance across the 4 sites. Limited dispersal through stepping-stone islands remains possible but unverified. Radu et al. (2024) suggest that the LEI silvereye population may have originated from the Capricorn or Bunker island groups (which includes HI), indicating a relatively recent shared ancestry that could also explain their vocal similarity.

### Spectral features

Findings also revealed unique traits in frequency characteristics in island dialects. Island populations showed a significantly higher mean frequency value in their songs than mainland populations. This finding contrasts with expectations based on previous research showing that island silvereyes are about 20% heavier than their mainland counterparts and would thus be expected to sing at lower frequencies due to physiological constraints (Potvin 2013). The unexpectedly higher mean frequency in island repertoires raises the question of what factors drive this differentiation between the 2 geographic settings.

Previous research has shown that silvereyes can modify the spectral characteristics of their songs almost instantaneously in response to environmental changes such as anthropogenic noise, demonstrating a high degree of short-term vocal plasticity (Potvin et al. 2011; Potvin and Parris 2012; Potvin and Mulder 2013). These changes occur on contemporary, generational timescales characteristic of cultural transmission rather than deep evolutionary timescales. Such plasticity aligns with the predictions of the acoustic adaptation framework (Hansen 1979), and may help explain differences in mean frequencies observed between island and mainland sites. Island silvereyes of HI and LEI might have evolved higher song frequency to adapt to the local soundscape, hence minimizing an overlap with conflicting call frequencies of other avian species. By doing so, they reduce the chances of confusion and interference in communication, which can help them attract mates and establish territories. For example, research conducted on avian communities within an Amazonian rainforest revealed that species either selected specific times and locations for singing to minimize acoustic interference from other species, or evolved songs aimed at avoiding such interference (Luther 2009). White-capped noddies (Anous minutus) dominate the avian population on HI (Lesku et al. 2023) and LEI (Tidemann 2014). Noddy calls are thus a dominant feature of the soundscape of the islands in this study. While noddy bioacoustics has not been scientifically described, the mean frequency of their calls sits at around 3,748 Hz (Xeno-canto Foundation 2024). Consequently, it may be that island silvereyes adjust their vocalizations to avoid masking by noddies (3,959 Hz, compared with 3,788 Hz for mainland silvereyes) and thus maximize sound transmission. This adaptation would be plausible within the evolutionary timeframe of the HI and LEI populations, since silvereyes have the ability to modify the frequency of their vocalisations instantly to adapt to the acoustic environment (Potvin and Mulder 2013). This may reflect a selection pressure favoring higher-pitched songs on these particular islands, aligning with the acoustic adaptation theory. A future comparative analysis of the soundscapes in both habitats, and an analysis of the frequency characteristics of the avian community of each habitat, could highlight potential acoustic patterns explaining why silvereyes sing at higher frequencies on these islands, despite their larger body sizes.

Extended syllable durations in island silvereyes may reflect adaptations to island environments. This aligns with the concept of island naiveté, where populations face reduced predation pressure and thus display less defensive behavior (Blumstein and Daniel 2005). The reduced need for vigilance could allow individuals to invest more time in sustained social signaling—such as territory defense or mate attraction—resulting in longer, more elaborate syllables. Indeed, syllables were significantly longer on islands compared with mainland sites. However, average slope values, representing syllable modulation, were similar across sites. The classification system also revealed a significant overlap in syllable types among sites. Together, these results indicate that island and mainland silvereyes share a common set of syllable structures, but island birds produce them at a slower articulation pace. This is consistent with previous observations of longer song duration in other island bird species (Morinay et al. 2013), a pattern potentially linked to the reduced predation pressures and different time budgets of insular life.

Multivariate analyses support these interpretations. A global PCA revealed that island syllables tend to occupy distinct regions of acoustic space, and a PERMANOVA on the Euclidean distance matrix detected a statistically significant, although small, effect of region on multivariate spectral structure. This confirms that island and mainland syllables occupy distinct regions of multivariate acoustic space. The loadings from the global PCA (see Supplementary Materials) suggest this divergence is primarily driven by traits such as frequency and duration, with island syllables trending towards higher and longer values. Importantly, Mantel tests showed no significant correlations between spectral, genetic, or geographic distance, suggesting that multivariate spectral divergence is shaped by local ecological or cultural processes rather than neutral evolutionary history.

### Dialect similarities

A high degree of similarity among the 4 dialects was observed. Strong similarities in the distribution pattern of syllables were observed at all levels of classification (L0, L1 and L2). However, this consistency does not necessarily indicate a cultural exchange between the 4 populations, as it could also arise from certain shared environmental pressures shaping dialects. To further understand the underlying mechanisms driving these similarities, the significance of specific syllables used in silvereye communication could be explored in future studies. This could involve conducting playback experiments and observing behavioral responses to elucidate their role in social interactions.

We also examined how similarly syllable groups are used across sites using multiple statistical approaches. These results demonstrate a nuanced pattern of cultural consensus: populations show strong overall agreement in syllable rankings yet substantial local variation in the precise ordering of middle-ranking syllables. This suggests that while certain syllable types are consistently prioritized across populations (likely reflecting structural or functional constraints), there is considerable flexibility in how less extreme syllables are utilized, consistent with cultural drift and local innovation. Histograms illustrate these long-tailed usage patterns, supporting the conclusion that dialects share core elements but differ in how these elements are deployed.

Finally, Mantel tests using both syllable-composition distances and spectral distances showed no statistically significant correlations with either genetic or geographic distance. Thus, dialects of the 4 populations are not constrained or driven by the genetic signature of the 2 subspecies or by their geographic distance. However, our analyses cannot fully disentangle historical constraints (eg, founder effects or inherited standing variation) from post-colonization divergence or more recent cultural transmission processes, and our interpretation therefore focuses on the processes that can be evaluated from contemporary acoustic patterns. This mirrors findings in satin bowerbirds (Nicholls et al. 2006), where acoustic divergence did not align with genetic distance, and supports the interpretation that the evolution of silvereye dialects represents true cultural evolution. One limitation of our sampling design is that we did not include the closest mainland coastal populations to HI and LEI (eg, the Gladstone region). It is therefore possible that unsampled mainland sites share more similar dialects with the islands than FC and SC do. Our conclusions about island–mainland contrasts therefore apply specifically to the 4 populations sampled here. Future work including these intermediate coastal sites could better resolve how dialect similarity decays with distance from islands.

Although Radu et al. (2024) also provide estimates of asymmetric migration among these populations, the small number of population pairs (*n* = 6) severely limits the statistical power of correlations involving migration rates. For this reason, we restricted our formal tests to F_ST_ and straight-line geographic distance, interpreting acoustic patterns within the broader context of known migration dynamics. Future work incorporating additional mainland and intermediate coastal sites would allow migration-based models to be applied more robustly.

## Conclusion

The absence of correlation between acoustic distance and genetic or geographic distances across the 4 populations indicates that dialect divergence at this spatial scale is more strongly shaped by cultural evolution than by neutral population history or isolation-by-distance. Furthermore, similarities were observed between the 4 populations’ dialects, with comparable syllable distribution patterns. Interestingly, island sites shared the most repertoire similarities and exhibited greater syllabic diversity than the mainland sites, suggesting that insularity may promote distinctive cultural trajectories. Despite their larger body size, island populations also produced vocalizations with marginally higher mean frequencies and longer syllable durations than mainland populations, consistent with the possibility of habitat-specific acoustic adaptation acting through cultural pathways rather than genetic divergence. These findings refine, rather than contradict, previous expectations about dialect evolution on islands. They highlight that cultural drift, innovation, and local ecological conditions can shape vocal divergence independently of genetic structure, even over relatively small geographic scales. The sensitivity of dialect evolution to environmental factors highlights the potential vulnerability of these populations to habitat disturbances or environmental change, making it crucial for conservation efforts to consider not only genetic and demographic factors but also the cultural aspects of avian behavior.

## Supplementary Material

arag032_Supplementary_Data

## Data Availability

Analyses reported in this article can be reproduced using the data provided by Robert et al. (2026).
