# Supplementary material for: Island-mainland song divergence is decoupled from genetic and geographic distance in silvereyes
Source: Behav Ecol. 2026 Apr 9;37(3):arag032. doi: 10.1093/beheco/arag032 (PMC13501412; doi:10.1093/beheco/arag032)
Supplement: Supplementary file 1 [file arag032_supplementary_data.zip › Supplementary Material.docx]

**Supporting information: Supplementary Materials (1-12)**

**Supplementary Material 1**

Example spectrogram of a silvereye (*Zosterops lateralis*) song displayed in RavenPro 1.6. The light blue rectangle shows a manually selected song segment, and the red rectangle highlights a single syllable for which RavenPro extracts the full set of spectral features. Spectrograms are visualised with time (s) on the x-axis and frequency (kHz) on the y-axis, allowing measurement of key acoustic variables such as low and high frequency, peak and centre frequency, quartile frequencies, delta time, slope, and bandwidth. This schematic example illustrates the procedure used to isolate individual syllables and obtain acoustic measurements for subsequent analyses.

**Supplementary Material 2**

Visual representation of the syllable grouping system developed in this study with a description of each level of classification, repertoire size, and its intended use in the comparison of silvereye vocalisations. Each classification tier incorporates examples of spectrographic representations of silvereye syllables identified in this research, along with their categorisation based on visual resemblances.

| **L0** |   Repertoire of 254 different structures identified. Each syllable contains between 1 and 455 combined occurrences across the four sites. This represents the most detailed level of syllable classification, and captures differences in individual accents. Use of L0 is intended for the analysis of syllable diversity measures of individual accents. |
| --- | --- |
| **L1** | Group 1 \| Group 2 \| Group 3 \| Group 4 \| Group 5 \| Group 6;  \|  \|  \|  \|  \|  Repertoire of 46 different structures identified. Each group contains between 18 and 1129 combined occurrences across the four sites. This level of classification captures differences in dialects. Use of L1 is intended for the comparative analysis of silvereye dialects. |
| **L2** | Category 1 \| Category 2 \| Category 3;  \|  \|  Repertoire of 14 different structures identified. Each category contains between 123 and 1776 combined occurrences across the four sites. This level of classification captures generic shape patterns. L2 was used only descriptively, not in any statistical tests. |

**Supplementary Material 3**

Description of the six dataset groups used in this study. The datasets are organised into Excel files and were obtained from syllable extraction and classification in RavenPro, and from the raw syllable occurrence data.

| **Dataset** | **Description** |
| --- | --- |
| L0Occ, L1Occ, L2Occ | Syllable occurrence **count** (raw data) for all four sites. Dataset created for each level of classification (L0, L1, L2). |
| L0Perc, L1Perc | Syllable occurrences for all four sites converted into **percentage values** (proportion). Dataset created for levels of classification L0 and L1. |
| L0Bin, L1Bin | Syllable occurrences for all four sites converted into **binary values** (presence-absence). Dataset created for levels of classification L0 and L1. |
| L1Adj | Syllable occurrences for all four sites, **adjusted** to account for sample size differences: zero-value syllables were removed from each smaller sample of each 6 site pairs (percentage values) at classification level L1. |
| L1AdjBis | Syllable occurrences for all four sites, **adjusted** to account for sample size differences: zero-value syllables were removed only from the smallest sample of four sites (count of occurrences) at classification level L1. |
| Acoustic | Dataset combines spectral characteristic (LF, HF, CF, DT, PF, Sl, F25, F75, MF, Bw), syllable levels of classification (L0, L1, L2), track name, site (FC, HI, LEI, SC), and habitat type (Island/Mainland). |

**Supplementary Material 4**

Source for all datasets. Datasets used for these analyses are provided at:

<https://github.com/Anonymous9452/Zosterops-lateralis>

**Supplementary Material 5**

Description of the ten variables used to compare vocalisations between island and mainland silvereye populations. Definitions are sourced from the RavenPro 1.4 User’s Manual (2010), with the exception of mean frequency and bandwidth which were computed manually.

| **Song variable** | **Unit** | **Acronym** | **Definition** |
| --- | --- | --- | --- |
| High Frequency | Hz | HF | The upper frequency bound of the selection. |
| Low Frequency | Hz | LF | The lower frequency bound of the selection. |
| Peak Frequency | Hz | PF | The frequency at which max power occurs within the selection. |
| Centre Frequency | Hz | CF | The frequency that divides the selection into two frequency intervals of equal energy. |
| Delta Time | s | DT | The difference between a selection start time and end time. |
| Average Slope | Hz/ms | Sl | Rate of change of the frequency over time within the selection. |
| Frequency 25% | Hz | F25 | The frequency that divides the selection into two frequency intervals containing 25% and 75% of the energy in the selection. |
| Frequency 75% | Hz | F75 | The frequency that divides the selection into two frequency intervals containing 75% and 25% of the energy in the selection. |
| Mean Frequency | Hz | MF | Sum of low and high frequency, divided by two. |
| Bandwidth | Hz | Bw | The difference between high frequency and low frequency. |

**Supplementary Material 6**

Mean values per site for all spectral characteristics investigated in this manuscript.

| Average slope (Hz/ms) | Delta time (s) | Low frequency (Hz) | High frequency (Hz) | Centre frequency (Hz) |
| --- | --- | --- | --- | --- |
| FC: -0.06837135  HI: -1.62501685  LEI: -1.82077107  SC: -2.14763511 | FC: 0.1023488  HI: 0.1408292  LEI: 0.1252416  SC: 0.1096032 | FC: 3037.965  HI: 3192.805  LEI: 3137.387  SC: 3118.729 | FC: 4318.722  HI: 4729.615  LEI: 4776.983  SC: 4677.954 | FC: 3697.512  HI: 3878.304  LEI: 3916.941  SC: 3874.708 |
| Peak frequency (Hz) | Frequency 25% (Hz) | Frequency 75% (Hz) | Bandwidth (Hz) | Mean frequency (Hz) |
| FC: 3716.173  HI: 3873.316  LEI: 3951.180  SC: 3893.659 | FC: 3507.531  HI: 3673.353  LEI: 3705.698  SC: 3658.302 | FC: 3884.154  HI: 4086.830  LEI: 4139.954  SC: 4109.988 | FC: 1280.757  HI: 1536.81  LEI: 1639.595  SC: 1559.225 | FC: 3678.344  HI: 3961.21  LEI: 3957.185  SC: 3898.341 |

**Supplementary Material 7**

Correlation heatmap of the ten spectral features measured across all syllables (mean frequency (MF), bandwidth (Bw), average slope (Sl), frequency 75% (F75), frequency 25% (F25), peak frequency (PF), centre frequency (CF), high frequency (HF), low frequency (LF), and delta time (DT)). The matrix illustrates the degree of co-variability among spectral features (Pearson’s r), highlighting strong correlations between several parameters. These correlations demonstrate redundancy among variables and support the use of PCA to summarise multivariate spectral structure rather than interpret each feature independently.

**Supplementary Material 8**

Dissimilarity matrices representing the acoustic distance between silvereye populations site pairs, computed with the Gower index. Values closer to 1 indicate more distance, while values close to 0 indicate a higher degree of similarity. Datasets represented in the tables are (a) presence-absence (binary values) of syllables for each site (dataset L1Bin); (b) proportion (%) of syllable occurrences per site (dataset L1Perc); (c) proportion (%) of syllable occurrences per site with adjusted values, excluding zero-values from smaller sample of each site pair (dataset L1Adj).

**Supplementary Material 9**

Analysis of variance (ANOVA) results for all spectral characteristics investigated in this manuscript.

**ANOVA results per site (FC, HI, LEI, SC)**

Average slope

|  | Df | Sum Sq | Mean Sq | F value | Pr(>F) |
| --- | --- | --- | --- | --- | --- |
| Group | 3 | 1588 | 529.2 | 6.834 | 0.000135 |
| Residuals | 10548 | 816780 | 77.4 |  |  |
|  |  |  |  |  |  |

Delta time

|  | Df | Sum Sq | Mean Sq | F value | Pr(>F) |
| --- | --- | --- | --- | --- | --- |
| Group | 3 | 2.31 | 0.769 | 236.5 | <2e-16 |
| Residuals | 10548 | 34.3 | 0.0033 |  |  |
|  |  |  |  |  |  |

Low frequency

|  | Df | Sum Sq | Mean Sq | F value | Pr(>F) |
| --- | --- | --- | --- | --- | --- |
| Group | 3 | 17510000 | 5835865 | 25.98 | <2e-16 |
| Residuals | 10548 | 2370000000 | 224644 |  |  |
|  |  |  |  |  |  |

High frequency

|  | Df | Sum Sq | Mean Sq | F value | Pr(>F) |
| --- | --- | --- | --- | --- | --- |
| Group | 3 | 62790000 | 20930238 | 43.48 | <2e-16 |
| Residuals | 10548 | 5077000000 | 481348 |  |  |
|  |  |  |  |  |  |

Centre frequency

|  | Df | Sum Sq | Mean Sq | F value | Pr(>F) |
| --- | --- | --- | --- | --- | --- |
| Group | 3 | 12820000 | 4272403 | 22.66 | 1.29e-14 |
| Residuals | 10548 | 1989000000 | 188584 |  |  |
|  |  |  |  |  |  |

Peak frequency

|  | Df | Sum Sq | Mean Sq | F value | Pr(>F) |
| --- | --- | --- | --- | --- | --- |
| Group | 3 | 15670000 | 5224981 | 21 | 1.47e-13 |
| Residuals | 10548 | 2624000000 | 248808 |  |  |
|  |  |  |  |  |  |

Frequency 25%

|  | Df | Sum Sq | Mean Sq | F value | Pr(>F) |
| --- | --- | --- | --- | --- | --- |
| Group | 3 | 10870000 | 3622628 | 21.23 | 1.05e-13 |
| Residuals | 10548 | 1800000000 | 170651 |  |  |
|  |  |  |  |  |  |

Frequency 75%

|  | Df | Sum Sq | Mean Sq | F value | Pr(>F) |
| --- | --- | --- | --- | --- | --- |
| Group | 3 | 18580000 | 6193402 | 28.02 | <2e-16 |
| Residuals | 10548 | 2331000000 | 221011 |  |  |
|  |  |  |  |  |  |

Bandwidth

|  | Df | Sum Sq | Mean Sq | F value | Pr(>F) |
| --- | --- | --- | --- | --- | --- |
| Group | 3 | 35000000 | 11666121 | 25.1 | 3.53e-16 |
| Residuals | 10548 | 4902000000 | 464697 |  |  |
|  |  |  |  |  |  |

Mean frequency

|  | Df | Sum Sq | Mean Sq | F value | Pr(>F) |
| --- | --- | --- | --- | --- | --- |
| Group | 3 | 31400000 | 10466521 | 44.2 | <2e-16 |
| Residuals | 10548 | 2498000000 | 236822 |  |  |
|  |  |  |  |  |  |

**ANOVA results per habitat (island vs mainland)**

Average slope

|  | Df | Sum Sq | Mean Sq | F value | Pr(>F) |
| --- | --- | --- | --- | --- | --- |
| Group | 1 | 211 | 210.75 | 2.718 | 0.0993 |
| Residuals | 10550 | 818157 | 77.55 |  |  |
|  |  |  |  |  |  |

Delta time

|  | Df | Sum Sq | Mean Sq | F value | Pr(>F) |
| --- | --- | --- | --- | --- | --- |
| Group | 1 | 2.06 | 2.0616 | 629.6 | <2e-16 |
| Residuals | 10550 | 34.55 | 0.0033 |  |  |
|  |  |  |  |  |  |

Low frequency

|  | Df | Sum Sq | Mean Sq | F value | Pr(>F) |
| --- | --- | --- | --- | --- | --- |
| Group | 1 | 12590000 | 12590287 | 55.94 | 8.07e-14 |
| Residuals | 10550 | 2374000000 | 225067 |  |  |
|  |  |  |  |  |  |

High frequency

|  | Df | Sum Sq | Mean Sq | F value | Pr(>F) |
| --- | --- | --- | --- | --- | --- |
| Group | 1 | 20660000 | 20655866 | 42.57 | 7.14e-11 |
| Residuals | 10550 | 5119000000 | 485250 |  |  |
|  |  |  |  |  |  |

Centre frequency

|  | Df | Sum Sq | Mean Sq | F value | Pr(>F) |
| --- | --- | --- | --- | --- | --- |
| Group | 1 | 1673000 | 1672968 | 8.823 | 0.00298 |
| Residuals | 10550 | 2000000000 | 189605 |  |  |
|  |  |  |  |  |  |

Peak frequency

|  | Df | Sum Sq | Mean Sq | F value | Pr(>F) |
| --- | --- | --- | --- | --- | --- |
| Group | 1 | 191700 | 191729 | 0.766 | 0.381 |
| Residuals | 10550 | 2640000000 | 250228 |  |  |
|  |  |  |  |  |  |

Frequency 25%

|  | Df | Sum Sq | Mean Sq | F value | Pr(>F) |
| --- | --- | --- | --- | --- | --- |
| Group | 1 | 2832000 | 2832337 | 16.53 | 4.83e-05 |
| Residuals | 10550 | 1808000000 | 171380 |  |  |
|  |  |  |  |  |  |

Frequency 75%

|  | Df | Sum Sq | Mean Sq | F value | Pr(>F) |
| --- | --- | --- | --- | --- | --- |
| Group | 1 | 104000 | 103987 | 0.467 | 0.494 |
| Residuals | 10550 | 2350000000 | 222720 |  |  |
|  |  |  |  |  |  |

Bandwidth

|  | Df | Sum Sq | Mean Sq | F value | Pr(>F) |
| --- | --- | --- | --- | --- | --- |
| Group | 1 | 993200 | 993198 | 2.123 | 0.145 |
| Residuals | 10550 | 4936000000 | 467832 |  |  |
|  |  |  |  |  |  |

Mean frequency

|  | Df | Sum Sq | Mean Sq | F value | Pr(>F) |
| --- | --- | --- | --- | --- | --- |
| Group | 1 | 16370000 | 16374777 | 68.74 | <2e-16 |
| Residuals | 10550 | 2513000000 | 238201 |  |  |

**Supplementary Material 10**

Comparison of spectral characteristics distinguishing island and mainland habitats in four silvereye populations in South East Queensland: (a) delta time, b) high frequency, (c) low frequency, (d) mean frequency, (e) average slope, (f) bandwidth. Line inside the box represents the median. The box extends from the first quartile to the third quartile, indicating the interquartile range; dots above and below the box represent outliers; whiskers extend to the smallest and largest non-outlier values. Hollow circles denote outlier values.

**Supplementary Material 11**

Eigenvalues, variance percentage (%) and factor loadings of five variables following principal component analysis (PCA) for silvereye syllables. The variables with high positive correlation (> 0.8) are shown in bold and outlined. The variables with moderate positive correlation (between 0.5 and 0.8) are shown in bold. (a) loading values for mainland sites FC and SC (n=3,669); (b) loading values for island sites HI and LEI (n=6,883).

| (a) Mainland | (b) Island |
| --- | --- |
| \| PC1 \| PC2 \| PC3; Eigenvalue \| 1.856 \| 1.469 \| 1.027; % of variance \| 37.130 \| 29.383 \| 20.540; Bw \| 0.233 \| **0.854** \| -0.238; LF \| **0.855** \| -0.455 \| 0.237; DT \| 0.144 \| **0.721** \| 0.361; MF \| **0.976** \| 0.103 \| 0.079; Sl \| -0.313 \| -0.048 \| **0.882** | \| PC1 \| PC2 \| PC3; Eigenvalue \| 1.822 \| 1.389 \| 1.013; % of variance \| 36.443 \| 27.778 \| 20.264; Bw \| 0.034 \| **0.945** \| 0.043; LF \| **0.899** \| -0.313 \| 0.191; DT \| -0.424 \| 0.385 \| 0.488; MF \| **0.892** \| 0.396 \| 0.216; Sl \| -0.192 \| -0.306 \| **0.831** |

**Supplementary Material 12**

Supplementary Material 12 is provided as a separate Excel workbook containing statistical outputs for the analysis of syllable ranking consistency across sites. These analyses directly test whether populations agree on which syllable types are most versus least common, independent of differences in overall abundance.

Sheet 1: Summary of ranking consistency analyses

Sheet 2: Pairwise correlation matrix between sites

Sheet 3: Syllable proportion data by site

Sheet 4: Study design information
